# Knowing too little or too much: the effects of familiarity with a co-performer's part on interpersonal coordination in musical ensembles

**DOI:** 10.3389/fpsyg.2013.00368

**Published:** 2013-06-25

**Authors:** Marie Ragert, Tim Schroeder, Peter E. Keller

**Affiliations:** ^1^Music Cognition and Action Group, Max-Planck Institute for Human Cognitive and Brain SciencesLeipzig, Germany; ^2^Music Cognition and Action Group, The MARCS Institute, University of Western SydneySydney, NSW, Australia

**Keywords:** interpersonal coordination, body movement, music, ensembles, sensorimotor synchronization

## Abstract

Expert ensemble musicians produce exquisitely coordinated sounds, but rehearsal is typically required to do so. Ensemble coordination may thus be influenced by the degree to which individuals are familiar with each other's parts. Such familiarity may affect the ability to predict and synchronize with co-performers' actions. Internal models related to action simulation and anticipatory musical imagery may be affected by knowledge of (1) the musical structure of a co-performer's part (e.g., in terms of its rhythm and phrase structure) and/or (2) the co-performer's idiosyncratic playing style (e.g., expressive micro-timing variations). The current study investigated the effects of familiarity on interpersonal coordination in piano duos. Skilled pianists were required to play several duets with different partners. One condition included duets for which co-performers had previously practiced both parts, while another condition included duets for which each performer had practiced only their own part. Each piece was recorded six times without joint rehearsal or visual contact to examine the effects of increasing familiarity. Interpersonal coordination was quantified by measuring asynchronies between pianists' keystroke timing and the correlation of their body (head and torso) movements, which were recorded with a motion capture system. The results suggest that familiarity with a co-performer's part, in the absence of familiarity with their playing style, engenders predictions about micro-timing variations that are based instead upon one's own playing style, leading to a mismatch between predictions and actual events at short timescales. Predictions at longer timescales—that is, those related to musical measures and phrases, and reflected in head movements and body sway—are, however, facilitated by familiarity with the structure of a co-performer's part. These findings point to a dissociation between interpersonal coordination at the level of keystrokes and body movements.

## Introduction

Musical ensembles are able to produce coherently coordinated sounds with impressive accuracy and precision. Despite numerous factors that increase temporal uncertainty—such as noise in the performers' motor control systems, spontaneous expressive playing, and different interpretations and playing styles among co-performers—ensemble musicians manage to keep asynchronies between their sounds small and consistent; around the 30–50 ms range, on average (Shaffer, 1984; Rasch, 1988). In order to achieve such a high level of coordination among a group of individuals, ensemble musicians typically engage in varying amounts of joint rehearsal prior to performance. While such rehearsal obviously improves interpersonal coordination, the cognitive mechanisms underlying this learning process have not been extensively investigated in experimental studies. Nevertheless, descriptive work on rehearsal techniques and social interaction in ensembles (e.g., Ginsborg et al., 2006; Ginsborg and King, 2012) has identified two factors that seem to be essential for the improvement of coordination. One factor relates to an ensemble performer's degree of familiarity with the structural aspects of a piece of music, and the other relates to the performer's familiarity with expressive features of a particular artistic interpretation of the piece. The present article is concerned with the effects of these types of familiarity on the interpersonal coordination of body movements and sounds in musical ensembles.

### Musical structure and playing style

Gaining familiarity with structural aspects of a musical piece entails acquiring knowledge about its constituent features, including pitch patterns and rhythms. These features are typically arranged hierarchically (Lerdahl and Jackendoff, 1983). For example, individual tones are concatenated into melodic motives and phrases, which are then combined into larger sections within a piece's formal structure (Clarke, 1988; Thompson, 2009). Similarly, rhythms in much music can be defined relative to the temporal units of an underlying metric framework (i.e., hierarchically nested measures, beats, and beat-subdivisions) (London, 2004). Thus, a full description of musical structure will usually contain information at different hierarchical levels and time-scales.

Gaining familiarity with expressive aspects of a particular interpretation of a piece of ensemble music necessitates the acquisition of knowledge about co-performers' idiosyncratic playing styles. Playing style is determined by a performer's interpretative preferences and tendencies concerning the modulation of expressive performance parameters including tempo (musical speed) and dynamics (intensity changes) (Repp, 1990, 1992, 1998, 1999a,b; Gabrielsson, 1999; Keller, 2012). Aesthetically motivated deviations in local tempo are particularly germane to interpersonal coordination in ensembles because coordination will be good only to the extent that these deviations are matched across co-performers (Keller, in press). Expressive timing deviations can take place at time-scales ranging from micro-timing variations at the millisecond level, which influence the rhythmic character of a performance, to larger-scale accelerations and decelerations associated with tempo “rubato.” Timing deviations generally serve to highlight important points in musical structure (e.g., phrase boundaries) and to communicate a particular mood or feeling (Palmer, 1997).

Information about musical structure and co-performer playing style that is acquired through joint rehearsal is represented in the performer's memory. To the extent that rehearsal is collaborative, this collective knowledge can form the basis for a shared performance goal, that is, a common representation of the ideal ensemble sound across ensemble members (Ginsborg et al., 2006; Keller, 2008). Establishing a shared performance goal facilitates ensemble coordination by ensuring that co-performers plan to produce their parts in a manner that is mutually compatible (Keller, in press). The realization of these plans under the real-time constraints of performance is challenging, and ensemble musicians therefore develop systems of shared performance cues that assist in regulating and coordinating their actions (Ginsborg et al., 2006).

Performance cues are musical features that serve as “landmarks” in a mental map of a piece (Chaffin and Imreh, 2002). These landmarks may be linked to structural aspects of the music (e.g., phrase boundaries) and to expressive devices introduced by the performer (e.g., intensity changes, tempo changes, or specific micro-timing deviations such as delaying a certain tone). In ensembles, performance cues are defined not only with respect to one's own part, but also for co-performers' parts. Shared performance cues remind co-performers of shared performance goals, and provide features in the music's landscape to which co-performers jointly attend, and points at which they can plan to “meet” during a performance (Ginsborg and King, 2012). Shared performance cues thus facilitate interpersonal coordination when ensemble musicians are familiar with each other's parts and playing styles because in this case co-performers are guided by the same map.

Shared performance goals, plans, and cues residing in memory facilitate ensemble coordination by allowing co-performers to anticipate each other's action timing by generating online predictions. It has been proposed that such predictions are a consequence of the internal simulation of the action in question (Wilson and Knoblich, 2005; Schubotz, 2007). In musical contexts, a performer may experience action simulation phenomenologically in terms of anticipatory mental imagery for body movements and sounds (Keller, 2012). The predictive nature of action simulation (and its conscious manifestation as anticipatory imagery) arises due to its basis in the internal modeling of sensorimotor transformations between movements and their effects.

Work in computational movement neuroscience has identified two types of internal models within the action control system: forward and inverse (Wolpert et al., 1998). Forward models represent the causal relationship between motor commands and their effects on the body and environment: these models predict the consequences of executing a particular command. Inverse models represent transformations from intended action outcomes (sounds, in the case of music) to the motor commands that produce them: they allow an appropriate command to be selected ahead of time. The sensorimotor transformations represented by internal models are acquired and strengthened through active experience and observational learning (Wolpert et al., 2003; Schubotz, 2007; Cross et al., 2009).

Active experience leads to the development of forward and inverse models that, by running slightly ahead of movement, facilitate the planning and execution of one's own actions by allowing potential errors to be corrected before they occur (Wolpert et al., 1998). These internal models, along with a second class of model that develops through observational learning, can also be used to simulate others' actions in advance of their production (Wolpert et al., 2003; Keller, 2008). Such “socially endowed” internal models have the potential to facilitate interpersonal coordination in musical ensembles to the extent that the models accurately simulate co-performers' idiosyncratic playing styles (Repp and Keller, 2010). Results consistent with this claim have been obtained in a behavioral experiment in which pianists recorded one part of several duets, and then returned months later to play the complementary parts in synchrony with either their own or others' recordings (Keller et al., 2007). The finding that pianists synchronized best with their own recordings suggests that temporal predictions are most accurate when based upon internal models that simulate micro-timing variations in a manner that matches a co-performer's playing style.

Synchronizing with a recording of one's own performance is a special case of familiarity, because the cognitive system engaged in action simulation is the same system that produced the action in the first place. Another way in which familiarity may influence ensemble coordination is by affecting the specificity of the movements that are simulated. Neurophysiological studies on music and dance have revealed that regions of the brain that are involved in the execution of a particular action become activated when an individual watches or listens to someone else carrying out the action. Importantly, the strength and specificity of these brain activations is modulated by the degree to which an individual is familiar with the observed action (Haueisen and Knösche, 2001; Calvo-Merino et al., 2006; Cross et al., 2006; D'Ausilio et al., 2006; Lahav et al., 2007). For example, regions of the primary motor cortex that represent specific fingers become especially active when pianists listen to music that would be played by those particular fingers (Haueisen and Knösche, 2001). Movements related to aspects of sound production on a musical instrument (such as a pianist's keystrokes) can be simulated only when these actions are in the observer's behavioral repertoire (e.g., when a pianist sees or hears another pianist). In the absence of such specific knowledge, simulation is limited to more general, instrument-independent ancillary movements, such as a performer's body sway and head gestures (Keller, 2008).

### Interpersonal coordination in ensembles

The aim of the present study was to investigate the effects of familiarity with the musical structure of a co-performers' part, as well as their playing style, on interpersonal coordination in piano duos. Previous work indicates that interpersonal coordination in musical ensembles takes place on multiple levels simultaneously (e.g., Goebl and Palmer, 2009; Keller and Appel, 2010). In the following, coordination at two levels (sounds and body movements), and the relationship of these levels to hierarchies in musical structure, is described. Then, potential functional benefits of enlisting multiple levels of coordination are considered, and, finally, the question of the degree to which coordination at different levels can be independent is discussed. This background motivates our hypotheses concerning the effects of familiarity with musical structure and playing style on ensemble cohesion.

At a basic level, ensemble musicians endeavor to coordinate their sounds in such a way that interpersonal timing is accurate and precise. In the case of piano duos, coordination is accurate to the extent that the keystrokes triggering the sounds of the two pianists' parts are correctly aligned in time, ensuring that the asynchrony between nominally synchronous keystrokes is small. Precision refers to how stable the temporal alignment of keystrokes is throughout a piece. Analyses of interpersonal keystroke asynchronies in piano duos have revealed that a range of factors affects the accuracy and precision of coordination. These include the performers' levels of musical ensemble skill, the complexity of the music, auditory feedback from the co-performer (but not necessarily visual feedback), and the compatibility of co-performers' playing styles in terms of expression and preferred tempo (Keller et al., 2007; Goebl and Palmer, 2009; Keller and Appel, 2010; Loehr and Palmer, 2011).

At another level, co-performers' ancillary body movements—including swaying of the torso and head motion—may become coordinated during ensemble play. Such movements serve basic functions related to regulating the timing of a performance and establishing leader-follower relations (Clarke and Davidson, 1998; Varni et al., 2010). Ancillary movements also provide a tool for communicating a musician's expressive interpretation and playing style to his or her co-performers and the audience (Davidson, 1994, 2001; Davidson and Correia, 2002; Williamon and Davidson, 2002; King and Ginsborg, 2011). Such movements are typically yoked to musical phrases and higher-order metric units (Davidson, 2009).

Keller and Appel (2010) investigated the relationship between interpersonal coordination at the level of keystrokes and body sway in seven pairs of pianists playing duet music. Keystroke timing was recorded on digital pianos, and body sway coordination was measured using a motion capture system that tracked the movement of a marker attached to each pianist's back. The degree of keystroke asynchrony was found to be low to the extent that the body sway movements of the pianist playing the primo (melody) part led the movements of the pianist playing the secondo (accompaniment) part. Thus, interpersonal coordination at short time-scales associated with instrumental movements (keystrokes that trigger sounds) was systematically related to coordination at longer time-scales associated with ancillary body movements.

Systematic relationships in the timing of events at different time scales may be a consequence of the hierarchical structure of music and its basis in body movement. Relevant research on dance has revealed that individuals represent different levels of metrical structure in different body parts when moving to music (Leman and Naveda, 2010; Toiviainen et al., 2010). Specifically, relatively low metrical levels (beats and beat-subdivisions) are reflected in the periodic movement of body parts that are low in mass and rigidity, and hence naturally oscillate rapidly, while higher metric levels (group of beats, or measures) are reflected in movements of body parts that have greater mass and rigidity. There is, furthermore, evidence that lawful relations in the timing of sounds and body movements linked to different metric levels assist in predicting tempo fluctuations in music. A study that required participants to synchronize finger taps with the beat in expressively and mechanically timed piano performances found that information about timing deviations at low metric levels in the expressive performances were used to generate predictions about event timing at higher metric levels (Rankin et al., 2009).

In ensemble performance, tight relations between interpersonal coordination at multiple levels and time scales may have functional benefits that derive from the enlistment of hierarchically nested internal models (cf. Shaffer, 1984; Pacherie, 2008, 2012). Multiple models may thus simulate short-range local goals and plans related to individual movements and sounds (or brief sequences of these) at one level, as well as long-range goals and plans concerning higher levels of musical structure at another level. For example, in the context of piano playing, one set of internal models may generate predictions about the timing of specific keystrokes, while another set of models may predict ancillary head motion and body sway linked to musical phrases and higher-order metric units.

Although systematically related, interpersonal coordination at different levels and time scales may be independent to some degree. Keller and Appel (2010), for example, found individual differences in the relation between interpersonal coordination at the level of piano keystrokes and body sway. While evidence for primo lead (i.e., the degree to which the primo player's actions are temporally ahead of the secondo player's actions) was found in keystroke asynchronies for all piano duos in their study, the tendency toward primo lead in body sway varied between duos. This suggests that coordination at the level of keystrokes and body sway was not perfectly correlated. Thus, measures of interpersonal action timing at short time scales (corresponding to instrumental movements and low metric levels) and long time scales (corresponding to ancillary movements and high metric levels or phrase structure) provide complementary indices of overall ensemble coordination that may be partially dissociated.

### Hypotheses

In the present study, we measured interpersonal keystroke asynchrony and body movement coordination across repeat performances (or “takes”) of piano duos under conditions where pianists had not met their partner, and either had or had not practiced their partner's part, before coming to the laboratory. Therefore, although the musical structure of the co-performer's part was known for half of the pieces, pianists presumably did not know exactly how their partner would play his or her part at the start of each set of takes.

In the experimental condition where pianists do not have the opportunity to practice their partner's part of the duo beforehand, the structure of this part is initially unfamiliar (and hence we refer to this as the “unfamiliar” condition). We assume that, in this case, each pianist develops performance goals, plans, and cues for their own part during private practice, whereas shared goals, plans, and cues that take both parts into account are not consolidated. Furthermore, internal models for simulating the co-performer's part may not be initially available when the part is unfamiliar. However, we assume that increasing exposure to the co-performer's part and playing style across takes will lead to the formation of shared performance goals, plans, and cues, and to the acquisition of internal models that generate temporal predictions based on simulations that are faithful to the co-performer's idiosyncratic style.

In the condition where pianists practice both parts of the duo beforehand (we refer to this as the “familiar” condition), the structure of the co-performer's part is familiar already prior to the first take. In this case, each pianist comes to the recording session with his own set of performance goals, plans, and cues, as well as joint internal models that are fit for simulating both parts. However, we assume that, because pianists do not know exactly how their partner will play their part before the first take, temporal predictions will initially be based on simulations that are imbued with the pianist's own playing style instead of the co-performer's style (see Keller et al., 2007). As internal models become calibrated to the co-performer's style across takes, there should be an increase in the degree to which simulations match the partner's performance, and temporal predictions will therefore become more accurate.

Based on the foregoing, we hypothesize that in the unfamiliar condition, where the structure of the co-performer's part and their playing style are initially unknown, interpersonal coordination at the level of keystrokes and body motion will initially be poor but will improve as familiarity with structure and style increases across takes. In the familiar condition, where the structure of the co-performer's part is known but their playing style is initially unknown, we hypothesize that three possible outcomes are plausible.

If the benefits of familiarity with the structure of the co-performer's part outweigh costs associated with initially not knowing their playing style, then interpersonal coordination will be relatively high at the first take and remain stable across takes. However, to the extent that familiarity with the co-performer's style is more influential than familiarity with their part, then interpersonal coordination will start out relatively low and improve across takes. Finally, familiarity with the co-performer's part and playing style may both be influential, albeit at different levels of interpersonal coordination. Therefore, interpersonal coordination at the level of keystrokes and body motion may be dissociated when familiarity with the co-performer's playing style increases across takes while familiarity with the structure of their part remains relatively constant. Specifically, the accuracy and/or precision of keystroke synchrony at short time scales may benefit from increasing familiarity with the co-performer's idiosyncratic micro-timing, while body movement coordination at longer time scales may remain stable because it is linked more closely to musical phrase structure and information at higher-level metric units, which are familiar from the outset.

## Methods

### Participants

Twenty pianists (mean age = 24.75 years; *SD* = 3.11 years; 12 females) participated in this study. The pianists were performance majors at local music schools or were studying music education at Leipzig University. They had played piano for 16.05 years on average (*SD* = 4.64), with a mean starting age of 8.7 years (*SD* = 3.5). All but one pianist had experience in ensemble playing, for example, in piano duos, chamber groups, bands, or orchestras (mean ensemble experience = 10.05 years, *SD* = 5.21). Pianists were randomly assigned to 20 pairs, with each participant playing in two different pairings with a pianist with whom they had not played together prior to the experiment. Pianists received a nominal fee in return for participation.

### Materials and apparatus

Eight piano duets from the nineteenth century Western music tradition were selected for use as materials (see Supplementary Material for Notated Scores of the Duets). Participants had not encountered the pieces, which were rather obscure, prior to the study. Each duet consisted of a primo part, which used a relatively high pitch range and usually contained the melody, and a secondo part, which occupied a lower pitch range and was primarily accompanying in function. The pieces represented a range of musical styles and were of intermediate technical difficulty. An excerpt of approximately 60–180 s (based on the composer's notated tempo indication) was chosen from each piece. Start and end points of excerpts were congruent with boundaries in the music's formal phrase structure. Excerpts had regular metric structures and no large-scale tempo changes or silent pauses that would be difficult to coordinate in the absence of visual cues. All excerpts nevertheless contained considerable rhythmic variation and the musical styles afforded expressive modulations of local tempo, lessening the likelihood that interpersonal synchrony would arise merely as a by-product of maintaining a steady tempo. The sheet music was prepared showing the excerpts without the piece's name or composer so that participants would not readily be able to search for the complementary part in the case of pieces for which they received only one part (none did so).

The instruments employed were two digital pianos (Yamaha Clavinova CLP150) that were set to the default sound (“grand piano 1”) with the volume dial fixed at the 12 o'clock position. The pianos were positioned with their keyboards facing one another. In order to record the identity and timing of piano keystrokes, the MIDI (Musical Instrument Digital Interface) output jack of each piano was connected to the same MIDI interface, which was connected via USB to a computer running MaxMSP 5.0 software under the Windows XP Professional operating system.

**Figure 1:**
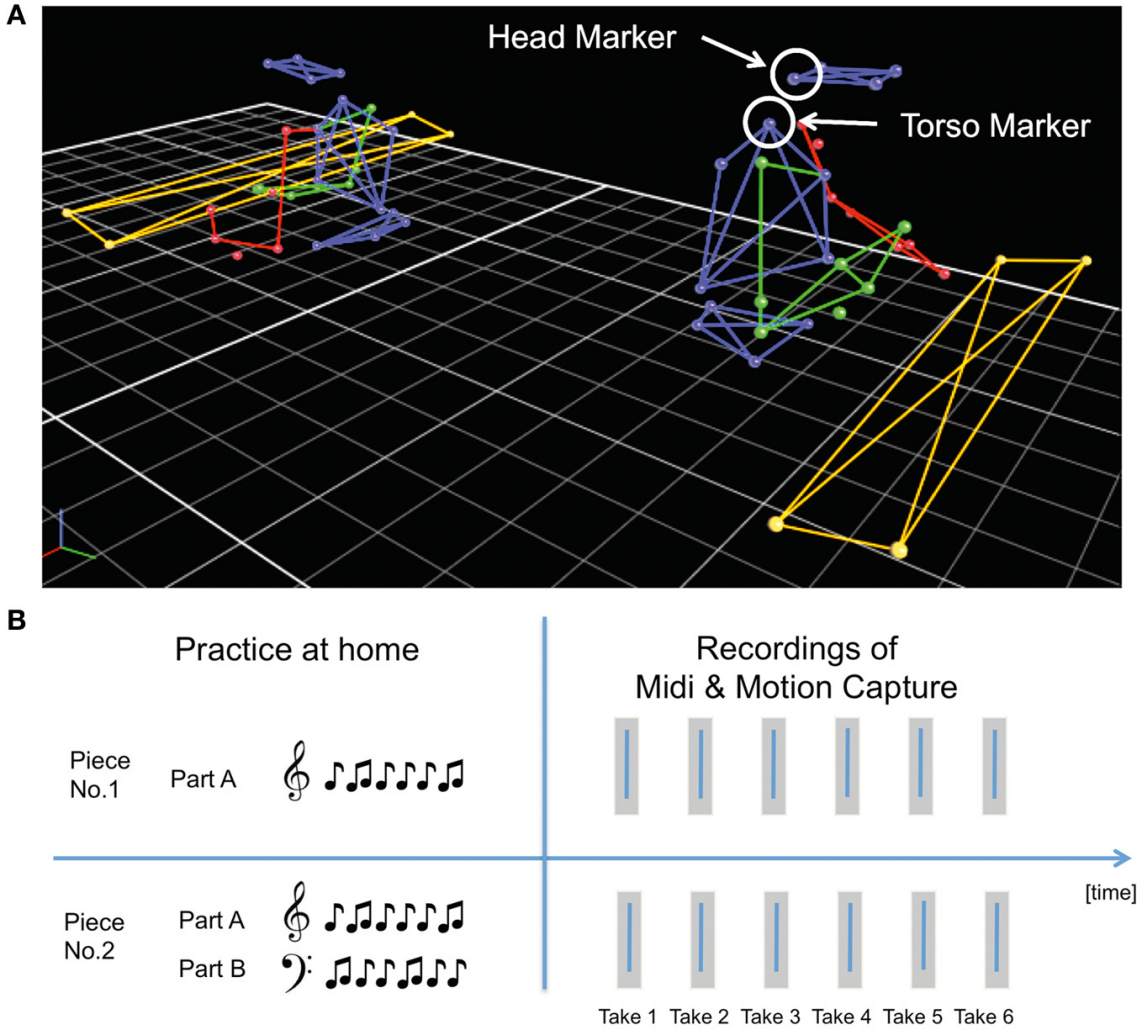
**(A)** Motion capture model representing the laboratory set up of pianos and placement of reflective motion capture markers on pianists' upper bodies. **(B)** Experimental design divided into two parts—practice at home and recordings of MIDI (keystroke) and motion capture data in the lab.

The movements of pianists' upper bodies were simultaneously recorded using a Vicon motion capture system. This entailed placing 25 reflective spherical markers (each 1.5 cm in diameter) on each of pianist's upper body in accordance with the Vicon plug-in-gait model (see Figure 1A). In addition, one marker was placed on the back of each hand near the index finger. Ten infrared cameras were used to track the position of each marker in 3D space with a 200 Hz sampling frequency. Motion capture data were recorded by Nexus software on a second Windows computer.

### Procedure

Out of eight piano duets, each pianist played four. As each pianist played in two different duo constellations (i.e., pairings), he or she played two pieces per duo, one per familiar and unfamiliar condition in each session. The procedure for each recording session—that is, each duo pairing—will now be described in detail.

Each pianist received sheet music for two duets 2 weeks prior to coming to the lab for each recording session (Figure 1B). For each duet they were also given the tempo indicated by the composer as a guide for the later recording sessions. The pianists were instructed to practice the pieces with a view to preparing polished performances. For one duet, they were asked to prepare both parts, so that the musical content of both parts was familiar when they entered the lab. For the second piece, participants only received one part (either the primo or secondo), so that only one's own part was known at the beginning of the recording session. The piece and part that each participant had to prepare was counterbalanced across participants, sessions and conditions. This manipulation resulted in different degrees of familiarity with the two duets at the beginning of each recording session. Thus, for the duet in one condition, each pianist was familiar with his or her own part but not the other's part; for the duet in the other condition, each pianist was familiar with both parts.

At the beginning of each session, pianists took turns at rehearsing their parts separately to get used to pianos and setting. While one pianist was practicing with headphones, anthropometric data were collected for the other pianist (these data were required for the motion capture plug-in gait model). Motion capture markers were then attached to their upper body. During the recording session, pianists sat at the pianos facing away from each other and could therefore not see each other while playing. This setup was adopted to isolate the effects of familiarity on coordination via auditory rather than visual information. Participants wore headphones through which they could hear the output of their own piano throughout the session and the co-performer's piano only during a take.

For the recordings, subjects were instructed to play their part as if it was a live performance. Six consecutive “takes” per piece in each condition were recorded. Note that pianists were not permitted to practice together prior to recording the takes or between takes, and thus only heard the co-performer's playing during each recording. At the start of each take, pianists were required to sit in a T-pose, i.e., holding their arms out at a 90° angle, and then to play some freely improvised tones on the keyboard for around 5 s. This preliminary procedure was necessary to create an active motion capture model with natural piano movements for each participant to be used later to label the markers semi automatically. To enable the pianists to start playing together at the instructed tempo in each take without visual contact, a metronome provided a lead-in by sounding for two bars (MIDI note number 98 or D7) before stopping and allowing the pianists continue autonomously. Pianists were asked not to stop playing during a take, even in the event of error. Performances were only interrupted and discarded if pianists could not continue due to catastrophic mistakes.

### Analysis of keystroke timing

Asynchronies between nominally synchronous keystrokes in the two parts of each duet were calculated for each of the recorded performances using the MIDI toolbox (Eerola and Toiviainen, 2004), for Matlab (MATLAB version 7.11. (R2010b) Natick, Massachusetts: The MathWorks Inc., 2010). In an initial step, a dynamic programming script (Large, 1993) was used to find matches between performed keystrokes and notes specified in the musical score. Onset times of keystrokes that matched the notation were extracted, and asynchronies between nominally simultaneous notes in both parts were calculated by subtracting the keystroke onset times of the secondo from the keystroke onset times of the primo part. The accuracy of interpersonal keystroke timing was indexed by computing the mean and the median of absolute (i.e., unsigned) asynchronies for each performance. Precision in interpersonal keystroke timing was indexed by calculating the coefficient of variation of (signed) asynchronies for each performance. The coefficient of variation was computed by dividing the standard deviation of asynchronies by the average inter-beat interval, a proxy for tempo, for each recording. The inter-beat interval was estimated by dividing the overall length of a recording by the number of beats it contained according to the musical score. As the variability of asynchronies typically increases with increasing inter-beat intervals, the coefficient of variation provides a measure of precision from which this general effect of tempo has been partialed out.

### Analysis of body movement coordination

Pianists' body movement data were preprocessed using Vicon Nexus software. In a first step all markers were automatically labeled, using the body models of the participants created at the beginning of the recording sessions. In a second step, gaps in the marker trajectories were manually detected and filled using the interpolation functions implemented in Nexus. Gaps occur when a marker is not detected by at least three Vicon cameras due to occlusion by the pianist's body or the piano.

The extracted movement data from each marker for each take were subsequently imported into Matlab for further analysis using the motion capture toolbox (Toiviainen and Burger, 2011). First, the raw position data were smoothed using a Savitzky-Golay filter with a window length of seven frames. Then the first two derivatives, velocity and acceleration, were calculated from the position data for each marker. For the following analysis, one marker on the head and another on the torso (see Figure 1A) were selected on each pianist, as these positions have been used successfully in previous work on piano duo coordination (Goebl and Palmer, 2009; Keller and Appel, 2010).

Interpersonal coordination of head and torso movements was quantified by computing mutual information. Mutual information is a non-linear measure of dependency based on the Shannon entropy (Shannon and Weaver, 1949; Cover and Thomas, 1991; Zahedi et al., 2010). For two random variables X and Y, the mutual information I is defined as, $I(X;Y)=\sum_{x\in X,\;y\in Y}P_{x,\;y}(x,y)\log_{2}\frac{P_{x,\;y}(x,y)}{P_{x}(x)\cdot P_{y}(y)}$ where *P*_*x*_ and *P*_*y*_ are marginal probabilities and *P*_*x, y*_ is the joint probability distribution of *X* and *Y*. Therefore, it can be thought of as a distance between the true joint probability distribution and the joint probability distribution of two independent random variables. In other words, the mutual information is the reduction in uncertainty, i.e., the gain in information, about one of the random variables after observing the other one. Mutual information is never negative, and only equal to zero if the two random variables are completely independent. It has recently been applied to quantify interdependencies, or coupling, between body movements (Schroeder et al., in preparation) and sounds (Papiotis et al., 2012, 2011) produced by interacting musicians. In the current study, the mutual information between corresponding markers on paired pianists was calculated to quantify body movement coordination for each take in the familiar and unfamiliar conditions.

To reduce the dimensionality of the data, the norm of the three-dimensional position, velocity and acceleration vectors was calculated, and the resulting normalized vectors were binned with a resolution of 1 mm, 1 mm/s, and 30 mm/s^2^, respectively. These values were chosen as a compromise, as too fine resolution requires substantially more data because the estimation bias for MI is approximately inversely proportional to the resolution. A too coarse resolution, on the other hand, might result in relevant information being discarded. In a final step, the MI between the two head markers and the two torso markers across members of each pair were calculated. The resulting values, in bits, represent the amount of information that the two markers share (i.e. a higher value indicating a strong dependence). Of interest here is not the absolute value of the amount of information shared, but the relative relationship for the two Familiarity and six Take conditions.

## Results

In the following, we will present results for interpersonal coordination in pianists' keystroke timing and body (head and torso) movement coordination, as well as the relationship between the keystroke timing and body movements.

Audio examples of a performance from take 1 in the familiar condition (Audio 1), take 6 in the familiar condition (Audio 2), take 1 in the unfamiliar condition (Audio 3), and take 6 in the unfamiliar condition (Audio 4) can be found in the Supplementary Material.

### Keystrokes

Two sets of analyses were run on keystroke timing. The first set focuses on the accuracy of interpersonal keystroke synchronization (as indexed by the median of absolute asynchronies) and performance tempo (as indexed by the average inter-beat interval). The second set of analyses focuses on a different measure of keystroke synchronization accuracy (mean absolute asynchrony) and on the precision of keystroke synchronization (indexed by the coefficient of variation). We examined two measures of keystroke synchronization accuracy (median and mean) because both are potentially informative. Median values are less strongly influenced by outliers than mean values, and hence provide a robust statistical measure of central tendency even when occasional large asynchronies are present in a performance. However, because such large asynchronies may be perceptually salient, they should not be ignored in analyses of music performance, and therefore mean absolute keystroke asynchrony is considered here. Data from six pairs of pianists were excluded from the analysis of keystroke timing for the following reasons. MIDI data of one pair had to be excluded due to a technical error during the recording session. Data from five additional pairs were classified as outliers because they contained values that were more than two standard deviations away from the grand average of the mean absolute keystroke asynchronies. Data from these pairs were excluded from analyses, leaving 14 pairs whose keystroke timing results will be reported in the following.

**Figure 2:**
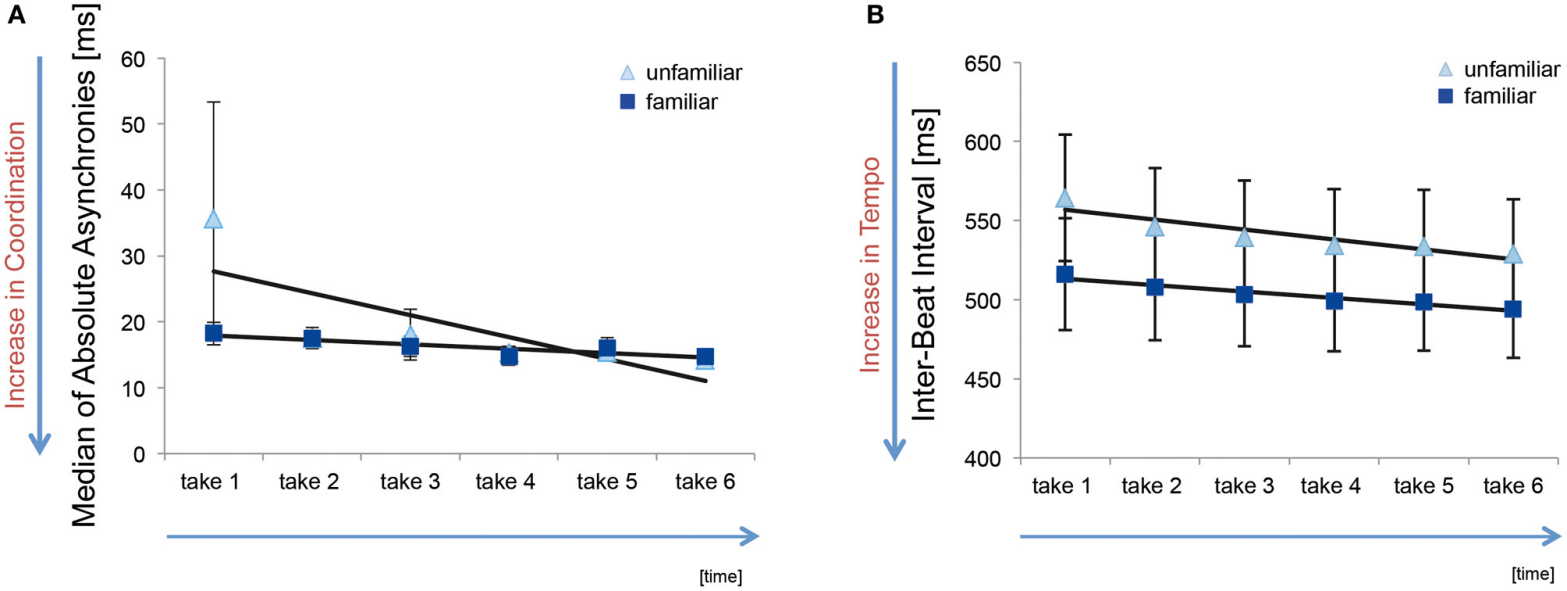
**(A)** Median of absolute keystroke asynchronies (in ms) across six takes in the familiar and unfamiliar condition. The light blue triangles represent the condition in which the co-performer's part was unfamiliar; the dark blue squares depict the condition in which both parts were known to both pianists. The black line shows a fitted linear trendline and the error bars show the standard error. **(B)** Inter-beat interval showing the effects of tempo.

The median of absolute asynchronies, averaged across pairs, for the six takes in the familiar and unfamiliar condition are shown in Figure 2A. The median values are within a range of 14–36 ms, which correspond to values found in previous studies of synchronization between skilled musicians in piano duos and other ensembles (see Keller and Appel, 2010; Keller, in press), and indicates overall accurate interpersonal coordination in terms of keystroke timing. Median asynchrony data were entered into a 2 × 6 repeated measures analysis of variance (ANOVA), with factors Familiarity (familiar vs. unfamiliar) and Take (1–6). The ANOVA yielded no significant effect of Familiarity, *F*_(1, 13)_ = 0.52, *p* = 0.48, η^2^_*p*_ = 0.038, Take, *F*_(5, 65)_ = 1.86, *p* = 0.12, η^2^_*p*_ = 0.125, or their interaction, *F*_(5, 65)_ = 1.07, *p* = 0.38, η^2^_*p*_ = 0.076.

Mean inter-beat interval data are shown in Figure 2B. An AVOVA on these data revealed a main effect of Take [*F*_(5, 65)_ = 14.272, *p* = 0.000, η^2^_*p*_ = 0.523], indicating an increase in tempo across repeat performances in both Familiarity conditions. The main effect of Familiarity on inter-beat interval, and the Familiarity × Take interaction, were not significant, *p* > 0.10.

**Figure 3:**
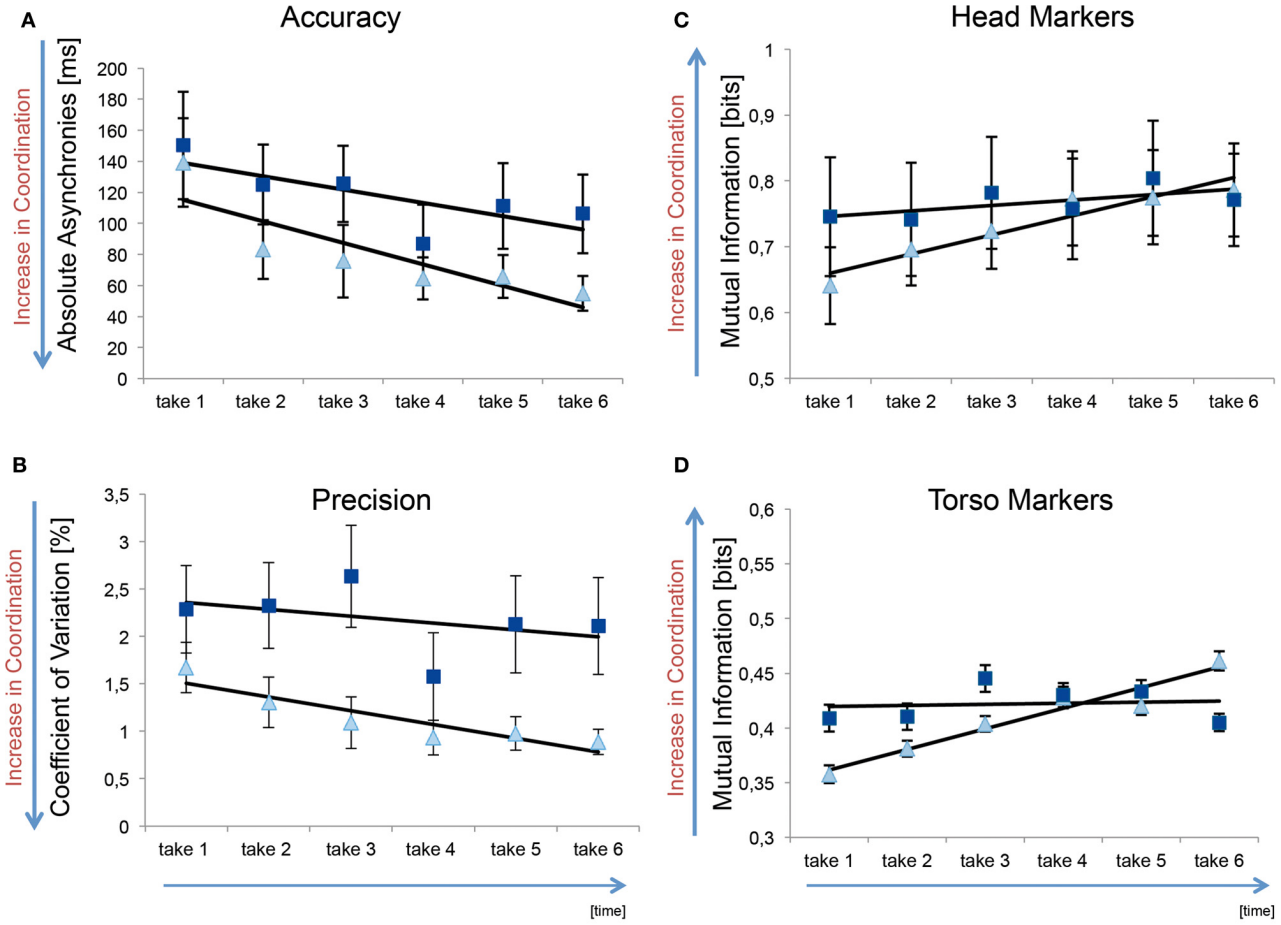
**(A)** Absolute keystroke asynchronies (in ms) across the six takes in the familiar and unfamiliar conditions. The unfamiliar condition is shown in light blue triangles, whereas the familiar condition is shown with dark blue squares. A linear trendline is shown in black. Error bars represent the standard error. A decrease in values indicates an increase in coordination **(B)** Coefficient of variation expressed as a percentage of the inter-beat interval. **(C)** Mutual information values (in bits) for the head markers. The light blue triangles represent the condition in which the co-performers' part was unfamiliar; the dark blue squares depict the condition in which both parts were known to both pianists. The black line shows a fitted linear trendline. The error bars show the standard error. An increase in mutual information indicates an increase in coordination. **(D)** Mutual information values for the torso markers.

Mean absolute asynchronies across the six takes in the familiar and unfamiliar condition are shown in Figure 3A. Here it can be seen that values for mean absolute asynchrony are generally higher than those for median absolute asynchrony (Figure 2A). This is due to the fact that means are more susceptible to the influence of occasional large asynchronies. The ANOVA on mean absolute asynchronies revealed a statistically significant main effect of Take, *F*_(5, 65)_ = 7.77 *p* = 0.002, η^2^_*p*_ = 0.374, but no significant main effect of Familiarity [*F*_(1, 13)_ = 1.57, *p* = 0.23, η^2^_*p*_ = 0.108] or interaction effect of Familiarity and Take [*F*_(5, 65)_ = 0.66 *p* = 0.66, η^2^_*p*_ = 0.048]. As can be seen in Figure 3A, mean absolute asynchrony decreased across takes similarly in the familiar and unfamiliar condition. This indicates a general improvement in synchronization accuracy across repeat performances. The fact that there was no analogous effect for median absolute asynchronies implies that the effect of Take on mean absolute asynchronies was related primarily to a reduction in the frequency of large asynchronies across repeats.

Data for the variability of keystroke asynchronies, indexed by the coefficient of variation, are shown in Figure 3B. As tempo increased across takes it was deemed necessary to analyze the coefficient of variation (rather than the variance or standard deviation of asynchronies) to test whether keystroke variability is affected by our manipulation independently of the increase in tempo. An ANOVA on the coefficient of variation data yielded significant main effects of Familiarity, *F*_(1, 13)_ = 5.202, *p* = 0.04, η^2^_*p*_ = 0.286, and Take, *F*_(5, 65)_ = 3.856, *p* = 0.00, η^2^_*p*_ = 0.229, but no significant interaction, *F*_(5, 65)_ = 1.086, *p* = 0.38, η^2^_*p*_ = 0.077. These results indicate that the variability of asynchronies was generally greater in the familiar than the unfamiliar condition, and decreased across repeat performances similarly in both Familiarity conditions.

To summarize, the analyzes of keystroke asynchronies suggest the accuracy of interpersonal coordination increased across repeat performances—primarily due to a reduction in large asynchronies—in a manner that was not influenced by whether the co-performer's part had been practiced beforehand. Although the precision of interpersonal coordination also increased across repeat performances, it was generally better when the co-performer's part had *not* been practiced beforehand.

### Body movement coordination

No technical issues or outliers prevented the inclusion of data from all 20 pairs of pianists in the analysis of the interpersonal coordination of body movements. Our detailed description of the results is limited to effects observed in mutual information data for velocity of the head markers and the torso markers, as this measure turned out to be more informative than measures of position and acceleration. However, for the sake of completeness, we display ANOVA results for position and acceleration of the head markers and the torso markers in Table 1 and averaged mutual information data for these variables in Table S1 in the Supplementary Material.

**Table 1:** **Results for the 2 × 2 × 6 (Familiarity × Marker × Take) ANOVA for position and acceleration data**.

**Motion capture measures *N* = 20**	**Main effect marker**	**Main effect initial familiarity**	**Main effect take**	**Interaction (marker × initial familiarity)**	**Interaction (take × marker)**	**Interaction (take × initial familiarity)**	**Interaction (take × initial familiarity × marker)**
Position	*F*_(1, 13)_ = 2.13, *p* = 0.17	*F*_(1, 13)_ = 0.65, *p* = 0.43	*F*_(5, 65)_ = 0.79, *p* = 0.56	*F*_(1, 13)_ = 0.82, *p* = 0.38	*F*_(5, 65)_ = 1.96, *p* =.097	*F*_(5, 65)_ = 1.43, *p* = 0.23	*F*_(5, 65)_ = 0.51, *p* = 0.77
Acceleration	***F*_(1, 13)_ = 27.09, *p* = 0.00**	*F*_(1, 19)_ = 0.4, *p* = 0.54	*F*_(5, 65)_ = 0.8, *p* = 0.56	*F*_(1, 13)_ = 0.00, *p* = 0.99	*F*_(5, 65)_ = 1.26, *p* = 0.29	*F*_(5, 65)_ = 0.83, *p* = 0.53	*F*_(5, 65)_ = 1.99, *p* = 0.09

Significant effects are printed in bold font.

Mutual information values for the velocity of head and torso markers, averaged across pairs, for the six takes in the familiar and unfamiliar condition are shown in Figures 3C,D. These data were analyzed in a 2 × 2 × 6 ANOVA, with the factors Marker (head vs. torso), Familiarity (familiar vs. unfamiliar), and Take (1–6). This analysis revealed statistically significant main effects of Marker [*F*_(1, 19)_ = 103.4, *p* = 0.00, η^2^_*p*_ = 0.845] and Take [*F*_(5, 95)_ = 3.25, *p* = 0.01, η^2^_*p*_ = 0.146], while the main effect of Familiarity was not significant [*F*_(1, 19)_ = 0.242, *p* = 0.63, η^2^_*p*_ = 0.013]. As can be seen in Figures 3C,D (please note the difference in scale on the vertical axes), mutual information—and therefore interpersonal coordination—was greater for head movements than for body sway. Although the main effect of Take suggests that mutual information generally increased across repeat performances, this effect was qualified by an interaction between Marker and Take [*F*_(5, 95)_ = 3.37, *p* = 0.01, η^2^_*p*_ = 0.151]: the increase in interpersonal coordination was steeper for head movements than body sway.

Most interestingly, the ANOVA revealed a significant interaction between Familiarity and Take [*F*_(5, 95)_ = 2.97, *p* = 0.02, η^2^_*p*_ = 0.135], but no significant three-way interaction [*F*_(5, 95)_ = 1.79, *p* = 0.12, η^2^_*p*_ = 0.086]. As can be seen in Figures 3C,D, mutual information in head movements and body sway started out high, and remained so across takes, in the familiar condition, while it started out relatively low and increased across takes in the unfamiliar condition. Separate analyses of data for the head markers and the torso markers confirmed that the interaction between Familiarity and Take was present both in head movements [*F*_(5, 95)_ = 2.5, *p* = 0.04, η^2^_*p*_ = 0.116] and in body sway [*F*_(5, 95)_ = 3.41, *p* = 0.01, η^2^_*p*_ = 0.152].

### Relationship between keystrokes and body movement coordination

The patterns of results reported above for keystroke asynchronies and mutual information in co-performers' body movements are qualitatively different from one another (as can be seen clearly in Figures 3A–D). This is suggestive of a dissociation between interpersonal coordination at the level of keystrokes and body movements. To test whether there is nevertheless some degree of overlap in these different measures of ensemble coordination (see Keller and Appel, 2010), we conducted a series of correlation analyses.

Keystroke asynchrony is a measure of error, and hence decreases with increasing coordination. Mutual information, on the other hand, is a measure of coupling, and hence increases as coordination increases. We therefore expected the two measures to be negatively correlated. Including data from only the 14 pairs that went into the keystroke analysis, we ran a set of partial correlation analyses. Data series that were entered into each analysis consisted of 168 data points (14 pairs × 2 familiarity conditions × 6 takes). Partial correlations were estimated between each measure of keystroke asynchrony and each measure of body movement coordination (position, velocity, and acceleration), controlling for the effects of Familiarity and Take. The results of these analyses are given in Table 2.

**Table 2:** **Partial correlations between keystroke and body coordination, controlling for the effects of Familiarity and Take (*df* = 164)**.

	**Midi measures**		
***N* = 14**	***Median absolute asynchronies***	***Mean absolute asynchronies***	***Coefficient of variation***
**MOTION CAPTURE MEASURES**			
***Head marker***			
Position	*r* = −0.09, *p* = 0.23	***r* = −0.35, *p* = 0.00**	***r* = −0.34, *p* = 0.00**
Velocity	*r* = −0.07, *p* = 0.35	***r* = −0.37, *p* = 0.00**	***r* = −0.39, *p* = 0.00**
Acceleration	*r* = −0.13, *p* = 0.10	*r* = −0.05, *p* = 0.52	*r* = −0.05, *p* = 0.50
***Torso marker***			
Position	*r* = −0.06, *p* = 0.44	***r* = −0.33, *p* = 0.00**	***r* = −0.31, *p* = 0.00**
Velocity	*r* = −0.05, *p* = 0.49	***r* = −0.27, *p* = 0.00**	***r* = −0.34, *p* = 0.00**
Acceleration	*r* = 0.07, *p* = 0.35	*r* = 0.01, *p* = 0.92	*r* = 0.05, *p* = 0.53

Significant correlations are printed in bold font.

**Figure A1:**
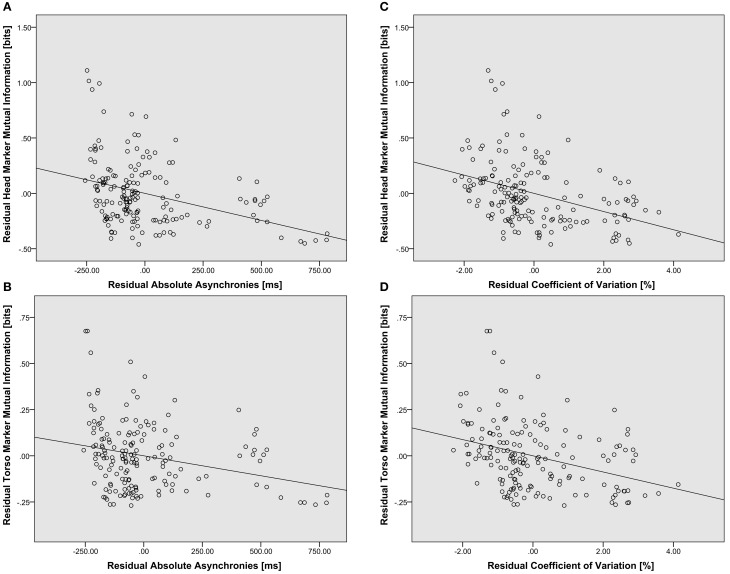
**Scatterplots showing partial correlations between measures of keystroke asynchrony (mean absolute asynchronies and coefficient of variation) and body movement coordination (velocity of head and torso markers), controlling for the effects of Familiarity and Take**. Each circle represents the residual value for a single duo performance (one pair on one take in one condition). **(A)** Partial correlation of head marker mutual information (in bits) and mean absolute keystroke asynchronies (in ms). **(B)** Partial correlation of torso marker mutual information and mean absolute asynchronies. **(C)** Partial correlation of head marker mutual information and the coefficient of variation in keystroke asynchronies (in %). **(D)** Partial correlation of torso marker mutual information and the coefficient of variation.

Significant negative partial correlations were found between mean absolute keystroke asynchrony and mutual information for head markers and torso markers (see Figure A1). This indicates that the accuracy of interpersonal coordination at the level of keystroke timing was systematically related to coordination at the level of head movements and body sway. These effects were not observed in an analysis of the relationship between median (rather than mean) absolute asynchrony and body movements. With regard to the precision of interpersonal coordination, significant negative partial correlations were found between the coefficient of variation and mutual information for head movements and torso movements.

Taken together, the results of these correlation analyses suggest that pairs of pianists who display accurate and precise interpersonal coordination in keystroke timing also display relatively tight interpersonal coupling of head movements and body sway. Therefore, the dissociation between interpersonal coordination at the level of keystroke timing and body movements reported above is only partial.

## Discussion

The present study investigated how familiarity with a co-performer's part and playing style influences interpersonal coordination at the level of keystrokes and body movements in piano duos. Familiarity was manipulated by requiring the pianists to play repeat performances (six takes) of duet music for which they had practiced only their own part or both their own and their co-performer's part before coming to the laboratory. The musical structure of co-performer's part was hence familiar at the start of the recording session when pianists had practiced both parts of the duets beforehand but not when they had practiced only their own part. The co-performer's playing style, however, was initially unknown in both conditions (as the pianists had not played together previously), but presumably became increasingly familiar across repeat performances.

Results pointed to a partial dissociation between the effects of familiarity with the co-performer's part and playing style on interpersonal keystroke asynchrony and body (head and torso) movement coordination. On one hand, the accuracy and precision of synchronization at the level of keystrokes was correlated with the coordination of head movements and body sway across pairs of pianists. This reflects co-dependence in finger and body movements, and indicates the convergent validity of the measures (see also Keller and Appel, 2010). On the other hand, the effects of repeated performance on the quality of interpersonal coordination when the co-performer's part was initially familiar or unfamiliar differed markedly for keystrokes and body movements. This partial independence is consistent with the notion that finger movements and body movements have different functions in music performance: instrumental vs. ancillary, respectively (Cadoz and Wanderley, 2000; Nusseck and Wanderley, 2009).

At the level of keystroke timing, the accuracy of interpersonal synchrony increased across repeat performances (due to a reduction in large asynchronies) irrespective of whether the co-performer's part had been practiced, while the precision of keystroke synchrony—which also increased across repeat performances—started out better, and remained so, when the co-performer's part was initially unknown. At the level of head movements and body sway, interpersonal coordination was consistently high from the outset when the co-performer's part was familiar, while coordination started out relatively low and increased across repeat performances when the co-performer's part was initially unknown. Therefore, for body movement coordination, it was helpful to know the co-performer's part, whereas such knowledge was apparently detrimental to keystroke synchrony. This dissociation suggests that familiarity with a co-performer's part has differential effects on interpersonal coordination at the different time scales along which body movements and keystrokes evolve.

### Body movement coordination (long time scales)

When both pianists practice both parts of a duet, each individual acquires knowledge about melodic and rhythmic information at multiple levels in the hierarchical structure of their own part and the co-performer's part. Furthermore, each pianist has the opportunity to develop his or her own set of hierarchically arranged performance goals, plans, and cues, as well as multilayered internal models for simulating both parts.

Our results for body movements suggest that structural knowledge about the co-performer's part was beneficial to interpersonal coordination at relatively long time scales associated with head motion and body sway. This supports the hypothesis that internal representations of structural information facilitate predictions about the timing of musical phrases and higher-level metric units (such as measures) to which such body movements are typically yoked (see Davidson, 2009). On this view, accurate predictions about when a co-performer will arrive at the next higher-order structural boundary allow the performer to time their own movements accordingly at the appropriate time scale (cf. Shaffer, 1984; Lee and Schoegler, 2009. Thus, knowledge about higher-order structural boundaries may function to provide cue points at which co-performers plan to “meet” (see Ginsborg and King, 2012). In typical ensemble scenarios, where co-performers are in visual contact with one another (and oftentimes an audience), body movements at long time scales serve to communicate expressive intentions and assist interpersonal coordination (Davidson, 1994, 2001; Davidson and Correia, 2002; Williamon and Davidson, 2002; King and Ginsborg, 2011). The present results, obtained in the absence of visual contact, show that the interpersonal alignment of body movements may be a natural feature of ensemble performance when co-performers are familiar with each other's parts.

The finding that—when the co-performer's part had been practiced beforehand—interpersonal coordination of head movements and body sway was relatively high from the first take, and did not improve across repeat performances, suggests that foreknowledge of the structure of the co-performer's part was sufficient for good coordination at long time scales. The fact that increasing exposure to the co-performer's playing style across repeats did not affect the coordination of head movements and body sway implies that knowledge of a co-performer's playing style was not influential upon interpersonal coordination at these long time scales. Thus, knowledge about the structure of a co-performer's part may trump knowledge of their playing style when it comes to achieving interpersonal coordination at the level of higher-order musical units such as phrases and measures.

The importance of foreknowledge about the structure of a co-performer's part for interpersonal coordination at long time scales is underscored by results obtained in the condition where the co-performer's part had not been practiced beforehand. In this case, performance goals, plans, and cues, as well as internal models that drive action simulation, are initially restricted to each pianist's own part. In accordance with the hypothesis that repeat performances lead to the acquisition of knowledge about the structure of the co-performer's part, the coordination of head movements and body sway started out relatively low and increased across takes in this condition. Thus, auditory exposure to the co-performer's part during the recording session gradually led to the formation of a representation of the part's structure. As this representation developed, it became increasingly effective as a basis for generating predictions about the timing of higher-order structural boundaries, and therefore the coordination of body movements at associated time scales improved.

It is possible that the improvement in interpersonal body movement coordination in this condition was attributable not only to increasing familiarity with the co-performer's part, but also to increasing familiarity with their manner of performing it. Results obtained by Keller et al. (2007) illustrated the beneficial effects of familiarity with the co-performer's playing style by showing that pianists synchronized more accurately and precisely with recordings of their own than others' performances. Note, however, that this effect of familiarity occurred at the level of keystroke micro-timing (body sway coordination was not relevant to the aims of the Keller et al. study), which may be more informative about a performer's idiosyncratic playing style than body movements at longer time scales. This conjecture is supported by our results for interpersonal keystroke coordination, which we discuss next.

### Keystroke coordination (short time scales)

The interpersonal coordination of keystroke timing takes place at the millisecond level, and requires expressive variations in micro-timing to be matched across co-performers (see Keller, in press). Interpersonal keystroke synchrony is hence reliant upon accurate online predictions about what sounds a co-performer will play at short time scales (in the order of hundreds of milliseconds) associated with beats and beat subdivisions, and how the co-performer will play these sounds in terms of micro-timing variations at even shorter time scales (tens of milliseconds).

The present finding that familiarity with a co-performer's part had a detrimental effect upon the precision of interpersonal keystroke synchrony (as indexed by the coefficient of variation) supports the hypothesis that foreknowledge about the structure of a co-performer's part, in the absence of knowledge about their playing style, leads to predictions about micro-timing variations that are based instead upon each pianist's own personal playing style. This interpretation of our findings rests upon the assumption that practicing both parts of a duet in private results in the music being learnt, and represented in memory, with each pianist's own idiosyncratic patterns of expressive micro-timing (Ginsborg et al., 2006; Keller, 2008). Furthermore, when paired with an unfamiliar co-performer, a pianist initially does not know exactly how this new partner will play his or her part, and hence online predictions about event micro-timing in the co-performer's part are based on action simulations that are imbued with the pianist's own playing style rather than the co-performer's style (see Keller et al., 2007). This is detrimental to interpersonal coordination at the level of keystrokes because individual differences in playing style lead to some degree of mismatch between predictions and actual events at short time scales.

Our finding that the precision of interpersonal keystroke synchrony improved across repeat performances, however, suggests that this mismatch can be resolved rapidly. With increasing exposure to the co-performer's playing, pianists gradually gain knowledge about each other's idiosyncratic use of expressive variations in micro-timing. We assume that such learning proceeds via the calibration of internal models to the co-performer's style, leading to an increase in the degree to which simulations are faithful to the partner's performance. Predictions about the co-performer's keystroke timing therefore become more accurate across repeat performances. The finding that tempo increased across repeats (despite the presence of a lead-in metronome at the start of each take, and the fact that pianists were instructed to practice at home at the correct tempo) is consistent with the proposal that co-performers improved in their ability to predict each other's micro-timing variations, thus lessening uncertainty and the need to adopt cautiously slow tempi. Indeed, the observed increases in tempo may be generally indicative of improved online planning abilities (Drake and Palmer, 2000), specifically, joint planning abilities.

The fact that improvements in the precision of keystroke synchrony, and increases in tempo, across repeat performances were independent of whether the co-performer's part had been practiced beforehand (i.e., there was no statistical interaction between the factors Familiarity and Take) suggests that these learning effects are attributable to the acquisition of knowledge about the co-performer's playing style rather than the structure of their part. Thus, knowledge about a co-performer's playing style may trump knowledge about the melodic and rhythmic structure of their part when it comes to achieving interpersonal coordination at the level of keystroke timing in the millisecond range. Please note that even if co-performers are initially well matched in individual playing style, it would not necessarily be the match *per se* that leads to good coordination, but rather the fact that stylistic similarity allows each performer to generate accurate predictions about the other's timing (Keller et al., 2007; Keller, in press).

It is informative that the effects of familiarity with a co-performer's part and playing style described above were expressed most clearly in the precision of interpersonal synchrony (i.e., the variability of asynchronies). Results for the accuracy of synchronization—as indexed by the mean and median values of absolute asynchronies—were more nuanced. One noteworthy finding is that synchronization accuracy was not affected reliably by whether or not the co-performer's part had been practiced beforehand. This suggests that the size of interpersonal keystroke asynchronies was influenced neither by knowledge of the music's structure nor by each pianist having learnt both parts of the duet with micro-timing variations associated with their own playing style rather than their co-performer's style. As discussed earlier, practicing a co-performer's part may lead to a mismatch between predicted and actual event timing due to individual differences in style. The present findings suggest that such mismatches do not affect the absolute size of interpersonal keystroke asynchronies, but rather their variability. It may be the case that some events in the co-performer's part occur earlier than predicted, while other events occur later than predicted, resulting in a situation where each pianist produces keystrokes that vary in terms of whether they are early or late relative to their co-performer's keystrokes. This would yield a mixture of negative and positive asynchronies whose values are high in variability but not necessarily large in terms of mean or median absolute size.

Another noteworthy aspect of the results for synchronization accuracy is the difference observed for mean and median measures of absolute asynchrony: mean asynchrony was initially large and decreased across repeat performances, while median asynchrony was consistently small across repeats, and within the range encountered in ensemble performances under natural conditions (Shaffer, 1984; Rasch, 1988). This finding suggests that gaining familiarity with the co-performer's style led to a reduction in the frequency of occasional large asynchronies (which are more influential upon mean than median estimates). Such asynchronies may arise due to errors in keystroke timing or to gross failures in predicting a co-performer's timing. It is reasonable to assume that both of these sources of large asynchronies would decrease across repeat performances. The process by which these large asynchronies are “pruned” may involve the fine-tuning of action control parameters and predictions about the co-performer's playing style through the calibration of internal models.

## Conclusions

The present study revealed a partial dissociation between the effects of familiarity with a co-performer's part and playing style on interpersonal coordination at the level of keystrokes and body movements in piano duos. Our findings suggest that knowledge about the structure of a co-performer's part is especially important for the interpersonal alignment of ancillary body movements linked to the music's phrasal and metric structure at long time scales, whereas knowledge about a co-performer's playing style is necessary for the precise synchronization of keystrokes at short time scales associated with expressive micro-timing. Thus, the mechanisms that underpin interpersonal coordination in musical ensembles operate at multiple time scales that reflect the hierarchical structuring of both the music and the co-performers' action control systems.

We have proposed that these mechanisms may include multi-layered networks of internal models that simulate a co-performer's actions slightly ahead of their occurrence, thereby generating predictions about the time course of the co-performer's large-scale body movements as well as variations in the expressive micro-timing of sounds (Shaffer, 1984; Pacherie, 2008, 2012). The process of simultaneously generating predictions at multiple timescales may allow co-performers to achieve the blend of precision and flexibility that characterizes expert ensemble performance. Specifically, predictions at longer time scales may serve as a scaffold that anchors coordination, and, in so doing, grant the performers the opportunity to create novel stylistic interpretations of a piece by freely varying expressive performance parameters at shorter time scales. The development of multi-layered internal models, and the calibration of models at short time scales to co-performers' styles of playing, may be a hallmark of skill as an ensemble musician.

The partial dissociation between interpersonal coordination at the level of keystrokes and body movements was presaged by the results of an earlier study that addressed individual differences in leader-follower relations in piano duos (Keller and Appel, 2010). This earlier study found that, while the tendency toward primo lead in body sway varied between duos, evidence for primo lead was observed in signed keystroke asynchronies without exception across duos. Furthermore, the results of that study indicated that overall ensemble cohesion was associated with high congruence between leader-follower relations in keystrokes and body sway coordination. Other studies on ensemble performance have provided evidence that head movements also reflect leader-follower relations (Goebl and Palmer, 2009; Varni et al., 2010). Although the current study was not concerned with leader-follower relations, our finding that familiarity with the co-performer's part affected body sway and head movements raises the possibility that knowing the structure of a co-performer's part may facilitate the establishment of optimal leader-follower relations, and hence overall ensemble cohesion.

The observed dissociation between the effects of familiarity on interpersonal coordination at different levels of the action control hierarchy highlights the complementary nature of instrumental movements (finger keystrokes) and ancillary movements (head motion and body sway). Although measures of keystroke synchrony and body movement coordination may be systematically related—as seen in the present experiment and earlier studies (Goebl and Palmer, 2009; Keller and Appel, 2010)—neither measure alone provides a complete account of the quality of ensemble coordination. Each measure is best suited to indexing the quality of different aspects of ensemble coordination, and, furthermore, each measure has particular advantages and disadvantages from the standpoint of empirical research.

Measures of instrumental movements, such as finger keystrokes recorded on digital pianos, are directly related to perceived sounds, and, therefore, closely linked to the perception of synchrony in the auditory domain. However, the assessment of synchrony in instrumental movements may be thwarted by minor performance errors (potentially leading to major data attrition) or by uncertainty concerning which sounds the musicians intend to produce in synchrony (e.g., during joint improvisation). Moreover, as ensemble performance is often characterized by deliberate asynchronies (e.g., due to leader-follower relations) and differences in the perceptual onsets of sounds, it is difficult to establish a priori the degree of synchrony that should be considered to be optimal for a particular performance (Keller, in press). The measurement of co-performers' body movements circumvents these issues somewhat, plus it carries the advantage that body movements are readily comparable across performers of different instruments (because human bodies are more alike than instrument bodies). Nevertheless, the process of quantifying the coordination of musicians' body movements with tools such as motion capture technology and kinematic analysis (Wanderley et al., 2005; Keller and Appel, 2010; Varni et al., 2010) presents its own sets of challenges. Given the above considerations, it seems likely that a full understanding of how ensemble co-performers produce cohesive musical textures can best be achieved by exploring the interrelationship between interpersonal coordination at different time scales and levels of action control.

### Conflict of interest statement

The authors declare that the research was conducted in the absence of any commercial or financial relationships that could be construed as a potential conflict of interest.

## Supplementary material

Table S1 **Averaged Mutual Information data (SE) for position and acceleration of the head markers and the torso markers for the familiar and unfamiliar condition, across the six takes**.
