# Supplementary material for: Knowing too little or too much: the effects of familiarity with a co-performer's part on interpersonal coordination in musical ensembles
Source: Front Psychol. 2013 Jun 25;4:368. doi: 10.3389/fpsyg.2013.00368 (PMC3691551; doi:10.3389/fpsyg.2013.00368)
Supplement: Table S1 — Averaged Mutual Information data (SE) for position and acceleration of the head markers and the torso markers for the familiar and unfamiliar condition, across the six takes. [file DataSheet2.DOCX]

| ***Motion capture measures N=20*** | **Head Marker** | | | | | | **Torso Marker** | | | | | |
| --- | --- | --- | --- | --- | --- | --- | --- | --- | --- | --- | --- | --- |
|  | ***Take 1*** | ***Take 2*** | ***Take 3*** | ***Take 4*** | ***Take 5*** | ***Take 6*** | ***Take 1*** | ***Take 2*** | ***Take 3*** | ***Take 4*** | ***Take 5*** | ***Take 6*** |
| **Position** |  |  |  |  |  |  |  |  |  |  |  |  |
| **unfamiliar** | 1.338 (0.093) | 1.442 (0.103) | 1.429 (0.083) | 1.429 (0.089) | 1.520 (0.097) | 1.510 (0.107) | 1.383 (0.101) | 1.389 (0.094) | 1.399 (0.092) | 1.409 (0.094) | 1.4881 (0.106) | 1.496 (0.110) |
| **familiar** | 1.450 (0.104) | 1.447 (0.098) | 1.434 (0.113) | 1.449 (0.114) | 1.470 (0.104) | 1.526 (0.109) | 1.387 (0.100) | 1.397 (0.096) | 1.367 (0.115) | 1.379 (0.116) | 1.358 (0.110) | 1.440 (0.112) |
|  |  |  |  |  |  |  |  |  |  |  |  |  |
| **Acceleration** |  |  |  |  |  |  |  |  |  |  |  |  |
| **unfamiliar** | 0.736 (0.078) | 0.781 (0.083) | 0.791 (0.081) | 0.822 (0.082) | 0.827 (0.082) | 0.804 (0.079) | 0.416 (0.077) | 0.396 (0.060) | 0.394 (0.049) | 0.425 (0.060) | 0.420 (0.054) | 0.444 (0.060) |
| **familiar** | 0.813 (0.108) | 0.826 (0.106) | 0.843 (0.103) | 0.834 (0.096) | 0.883 (0.102) | 0.848 (0.105) | 0.387 (0.056) | 0.372 (0.056) | 0.402 (0.060) | 0.378 (0.052) | 0.388 (0.052) | 0.383 (0.054) |

Table S1: Averaged Mutual Information data (SE) for position and acceleration of the head markers and the torso markers for the familiar and unfamiliar condition, across the six takes.
