# Supplementary figures and images for: Knowing too little or too much: the effects of familiarity with a co-performer's part on interpersonal coordination in musical ensembles
Source: Front Psychol. 2013 Jun 25;4:368. doi: 10.3389/fpsyg.2013.00368 (PMC3691551; doi:10.3389/fpsyg.2013.00368)

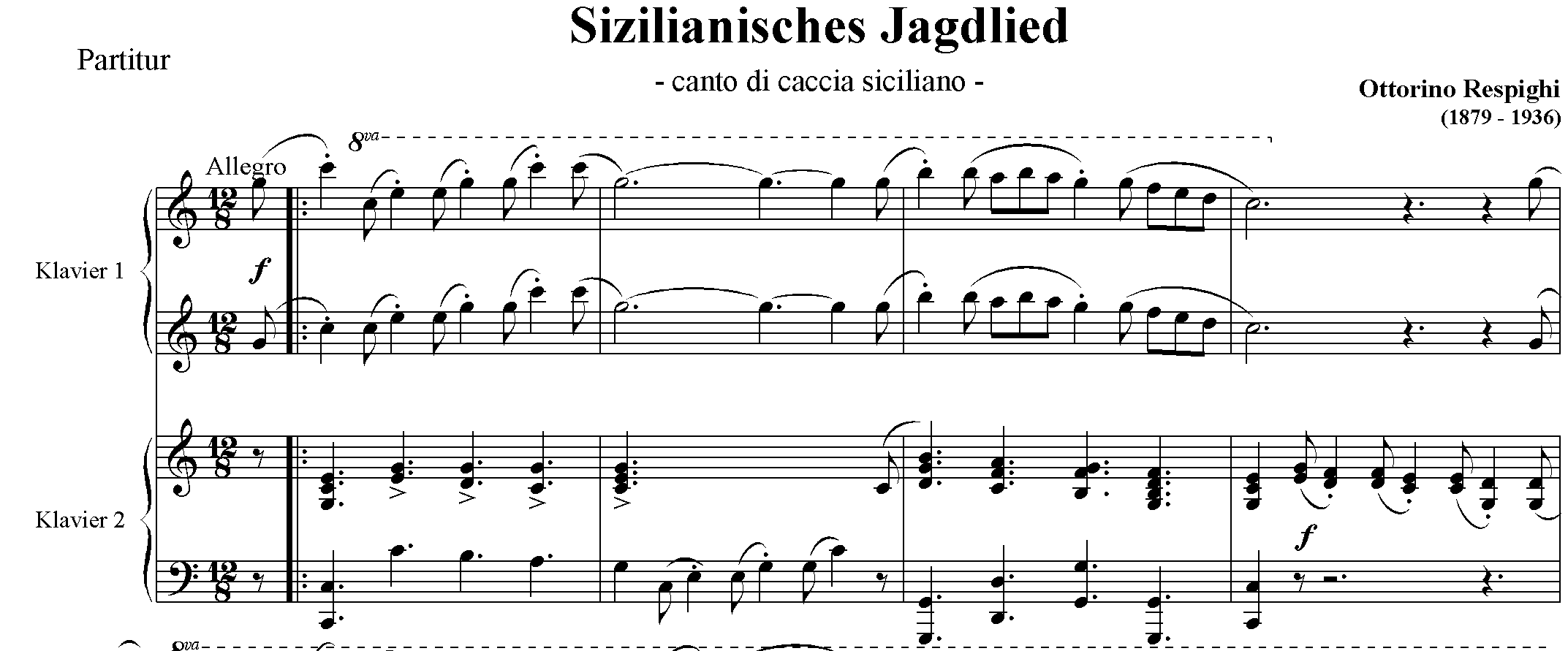

Supplement: Supplementary file 2 [file DataSheet1.ZIP › Fertige Notationen/Stueck7_1.TIF]
